# Cervical vestibular evoked myogenic potential asymmetry, but not amplitude, differentiates vestibular migraine during prolonged unidirectional visual motion

**DOI:** 10.3389/fneur.2026.1773410

**Published:** 2026-03-17

**Authors:** Elvira Cortese, Huseyin Nezih Ozdemir, Anca-Diana Grigore, Patricia Castro, Nehzat Koohi, Diego Kaski

**Affiliations:** 1 SENSE Research Unit, Department of Clinical and Movement Neurosciences, Institute of Neurology, University College London, London, United Kingdom; 2 Escuela de Fonoaudiología, Facultad de Medicina, Universidad de Valparaíso, Valparaíso, Chile; 3 Department of Neurology, Ege University Medical School, Izmir, Türkiye; 4 Faculty of Health and Life Sciences, School of Allied Health Sciences, De Montfort University, Leicester, United Kingdom; 5 Escuela de Fonoaudiología, Facultad de Medicina Clínica Alemana, Universidad del Desarrollo, Santiago, Chile

**Keywords:** biomarkers, cervical vestibular evoked myogenic potential, vestibular migraine, virtual reality, visual stimulation, visuo-vestibular interactions

## Abstract

**Introduction:**

Vestibular migraine (VM) is the leading cause of episodic vestibular complaints. It arises from altered brain states that disrupt sensory processing. A reliance on clinical history for diagnosis highlights the need for bedside biomarkers, particularly in emergency settings where misdiagnosis is common.

**Methods:**

In this cross-sectional study, 30 VM patients (median-age = 40.5; 27 females) from University College London vestibular clinics and 30 age-gender matched healthy controls (median-age = 32.5; 27 females) were recruited, between May 2024 and October 2025. Cervical Vestibular Evoked Myogenic Potential (cVEMP) responses were measured before and after a *Unidirectional Visual Motion Stimuli* delivered via virtual reality goggles.

**Results:**

Mixed linear modeling (MLM) showed no significant effects of group, condition or ear on cVEMP amplitude (all *p* > 0.05). MLM on asymmetry revealed a significant effect of group, *F*(1, 57.21) = 11.89, *p* = 0.001 and condition, *F*(1, 56.53) = 14.47, *p* = <0.001; but no significant group × condition interaction, *F*(1, 56.53) = 1.57, *p* = 0.215. Spearman correlations showed no association between cVEMP delta amplitude and DHI. VM patients scored higher on all symptom’s measures compared with controls (All *p* < 0.001).

**Conclusion:**

Prolonged unidirectional visual stimulation does not significantly affect cVEMP amplitude responses in VM, limiting its value as a differential diagnostic tool. The need to further explore asymmetry and interaural/interhemispheric sensory integration in VM is underscored.

## 1 Introduction

Vestibular migraine (VM) is the most common cause of non-positional spontaneous episodic vertigo, affecting between 1 and 2.7% of the population (1). VM is characterized by recurrent episodic vestibular symptoms, together with sensitivity to sensory stimuli, nausea, or vomiting. The vestibular symptoms can manifest as vertigo, dizziness, and/or imbalance, as well as misperceptions of self or environment motion and disorientation (2), lasting from minutes to days. Notably, headache is not always a prominent or defining feature of this condition (2).

VM results from an altered brain state (2) that may affect a distributed brain network involving vestibular regions, particularly in the brainstem and thalamocortical pathways (3). These alterations lead to a generalized sensory dysregulation that impairs the ability to process sensory inputs, regardless of whether they are visual, proprioceptive, or vestibular (2, 4). Because the vestibular system is multimodal, receiving afferent information from multiple systems, predominantly from vision and the proprioceptive system, such sensory dysregulation represents an obstacle to the construction of conscious and coherent perception and poses a major problem for VM patients in describing and/or localizing symptoms (2).

Diagnosing VM can therefore be complex (1, 5) as there are currently no specific quantitative markers for diagnosis. Indeed, the diagnosis is primarily based on clinical features and the exclusion of other conditions, as clinical investigations typically yield normal results. According to diagnostic criteria, a definitive diagnosis of VM requires a history of at least five episodes of vertigo or dizziness, with at least half of these episodes accompanied by classic migraine symptoms, such as headache, nausea, vomiting, and sensitivity to sensory stimuli (1). The current diagnostic criteria for VM inevitably introduce a delay in diagnosis (given the need for 5 or more episodes) that prevents timely counseling and treatment for affected individuals (6).

Moreover, VM is particularly challenging to diagnose in the acute stage, where the differential diagnosis includes stroke—that frequently share clinical features with VM (e.g., headache, central nystagmus or subtle ocular motor abnormalities)—and some chronic vascular conditions (i.e., Orthostatic hypofunction, small vessel diseases) (1, 7), leading to early VM spells becoming exceptionally challenging to diagnose in emergency settings. This situation often places additional pressure on emergency department staff who must prioritize the rapid triage of high-risk conditions but may lack the necessary tools to distinguish between VM and other central pathologies such as posterior territory stroke (8, 9). Consequently, there is a risk of stroke over-diagnosis placing undue burden on stroke services (10).

Therefore, there is a need to develop simple, bedside biomarkers to improve the early diagnosis of VM, in the early stages of the disease, precisely when a correct diagnosis of VM is most needed, to avoid unnecessary investigations and initiate preventative measures to reduce future attacks.

Advances in the understanding of the pathophysiological mechanisms of VM and the sensory distortions reported by VM patients (3, 11–13) have encouraged the exploration of novel biomarkers.

Visual stimulation (VS) capable of inducing visual vection has been shown to modulate cVEMP responses in healthy individuals, increasing response amplitudes (14, 15) and modulating latencies (16). These findings support the integration of visual input within vestibular reflex pathways, with effects that scale with the degree of visual immersion and are particularly pronounced in virtual reality paradigms.

In contrast, the effect of prologued unidirectional visual stimulation on vestibular reflexes remains largely unexplored. To date, no studies have specifically examined its impact on cVEMP responses in healthy humans. The closest evidence comes from Bednarczuk et al., who reported increased vestibulo-ocular and vestibulo-perceptual thresholds following prolonged unidirectional visual stimulation in individuals with vestibular migraine (11).

Converging neuroimaging data indicate altered metabolic activity in cortical and subcortical vestibular and visual regions in patients with VM during the ictal phase, further supporting the presence of an abnormal visuo-vestibular interaction in this condition (3).

Building on this evidence, the present study explores whether cervical vestibular evoked myogenic (cVEMP) responses alter following exposure to a unidirectional visual motion stimulus.

The *unidirectional visual motion exposure* paradigm has proven effective in modulating visual cortex activity in healthy subjects (17). This method has also shown similar application in individuals with bilateral vestibular failure (18), establishing it as a valid approach for investigating visuo-vestibular interactions. Moreover, cVEMP responses generate objective, non-invasive electrophysiological responses that may be more practical than evaluating perceptual threshold changes, following unidirectional visual motion exposure. Although cVEMPs are not yet standard bedside tools due to technical and interpretive requirements, emerging wireless and portable devices, such as the eVEMP or SmartVEMP systems (Biomed Jena; Intelligent Hearing Systems), now enable rapid assessment of vestibular reflexes even in hyperacute or emergency settings, highlighting their potential practical role in bedside evaluation and differential diagnosis of vestibular migraine.

## 2 Materials and methods

### 2.1 Design and participants

A cross-sectional study using non-probabilistic sampling was followed. The Sample Size was determined pragmatically, based on feasibility and prior studies involving VM patients (5, 11). Given the exploratory design and lack of existing data on the effects of visual motion on cVEMP responses in VM, a formal power calculation was not feasible. A target of 30 participants per group was therefore chosen to allow estimation of group-level effects and variability, and to provide preliminary data to inform future studies.

We recruited 30 patients (median age = 40.5, age range = 19–64; 27 females and 3 males) diagnosed with VM, coming from UCLH vestibular clinics, between May 2024 and October 2025; and 30 age-gender matched healthy controls (HC) (median age = 32.5, age range = 24–56; 27 females and 3 males).

All patients had active VM (at least one attack in the past year) diagnosed by neuro-otology specialists (DK and NK) following the Barany consensus diagnostic criteria (1), presented normal audio-vestibular responses, no vision-related defects and no other confounding pathologies. Patients were assessed in the inter-ictal phase, although three patients were mildly symptomatic on the day of testing. Nine patients were taking pharmacological (preventive) treatment to reduce the frequency of their VM episodes (see Table 1). Studying individuals with clinically well-defined VM following these previous criteria provides an appropriate model for investigating central visuo-vestibular interactions while minimizing confounding effects from structural vestibular pathology.

**Table 1:** Vestibular Migraine patient clinic and demographic information.

No.	Gender	Age	Time since last episode (months)	VM preventive treatment	VM phase	Symptoms on VS	Symptoms forced to stop VS
1	Female	24	6	No	Inter-ictal	Yes	No
2	Female	29	5	No	Inter-ictal	Yes	No
3	Female	33	1	No	Inter-ictal	Yes	No
4	Female	32	3	No	Inter-ictal	Yes	No
5	Female	36	0	Flunarizine 50 mg	Inter-ictal	Yes	No
6	Female	40	8	Flunarizine 50 mg	Inter-ictal	Yes	No
7	Male	42	3	No	Inter-ictal	Yes	No
8	Female	64	2	No	Inter-ictal	Yes	No
9	Female	20	1	No	Inter-ictal	Yes	No
10	Female	50	4	Flunarizine 50 mg	Inter-ictal	Yes	No
11	Female	45	1	No	Inter-ictal	Yes	No
12	Female	37	0	Nortriptyline 50 mg	Inter-ictal	Yes	No
13	Female	33	0	No	Inter-ictal	Yes	No
14	Male	59	8	Nortriptyline 30 mg	Inter-ictal	Yes	No
15	Female	56	1	Flunarizine 10 mg	Inter-ictal	Yes	Yes
16	Female	42	0	No	Inter-ictal	Yes	No
17	Female	48	0	No	Inter-ictal	Yes	No
18	Female	56	0	No	Inter-ictal	Yes	No
19	Female	62	0	No	Inter-ictal	Yes	No
20	Female	55	0	Candesartan 8 mg	Inter-ictal	Yes	No
21	Female	60	0	No	Inter-ictal	Yes	No
22	Female	37	0	No	Inter-ictal	Yes	No
23	Female	19	0	No	Inter-ictal	Yes	No
24	Female	54	0	No	Inter-ictal	Yes	No
25	Female	59	0	Propranolol 80 mg	Inter-ictal	Yes	No
26	Female	25	8	Nortriptyline 50 mg	Inter-ictal	Yes	No
27	Female	36	0	No	Inter-ictal	Yes	No
28	Male	27	1	No	Inter-ictal	Yes	No
29	Female	41	2	No	Inter-ictal	Yes	No
30	Female	30	1	No	Inter-ictal	Yes	No

VM, Vestibular migraine; VS, Visual stimulation.

Healthy Controls were selected to provide a normative comparison for vestibular reflex responses under identical experimental conditions. The control group comprised adult healthy individuals, with no diagnosis of migraine (neither headache nor vestibular), and no other medical diagnoses.

After signing informed consent, participants were asked to complete self-assessment questionnaires for the subjective quantification of dizziness (Dizziness Handicap Inventory—DHI), motion-sickness (Motion Sickness Susceptibility Questionnaire—MSSQ), depression and anxiety (Hospital Anxiety and Depression Scale—HADS) and migraine (Headache Impact Test—HIT-6) (19–22).

### 2.2 cVEMP response recordings

**Figure 1:**
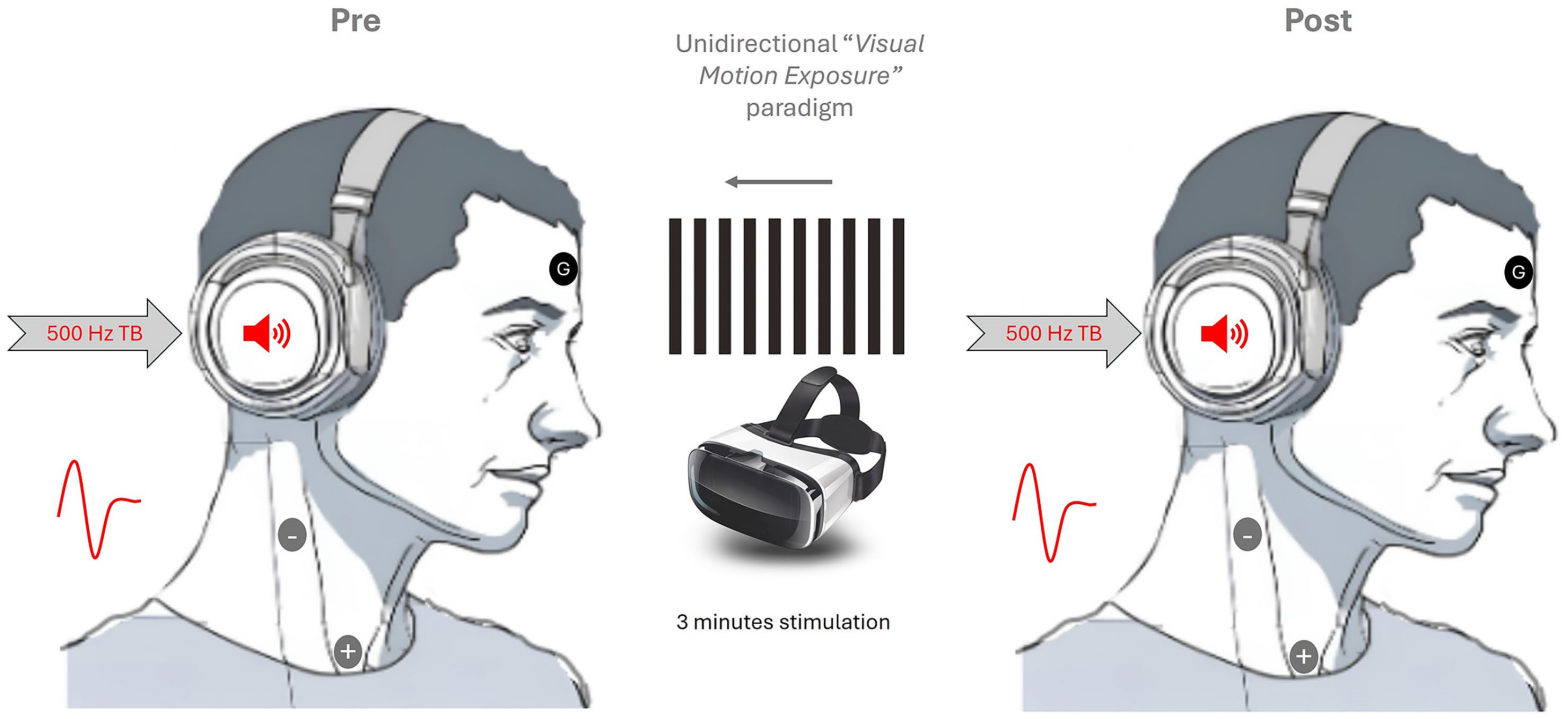
Experimental setup and paradigm.

Cervical Vestibular Evoked Myogenic Potential was assessed using an *Eclipse* platform (Interacoustics^®^) and following standard clinical procedure (23, 24). For cVEMP, the electrode montage consisted of a reference electrode (negative) placed at the sternocleidomastoid muscle (SCM) belly, a positive electrode placed on the sterno-clavicular junction, and a ground electrode placed on the forehead (Figure 1).

Participants were seated and asked to turn their heads in the opposite direction to the sound to ensure adequate SCM activation, which was controlled by monitoring the EMG activity and giving active feedback to the participants. Stimulation was delivered monaurally through air conduction (AC) TDH39 earphones and 500 Hz Tone Burst (TB) (rarefaction polarity), with a linear envelope (2-ms rise/fall time, 2-ms plateau, at a repetition rate of 5.1 per second, 200 averaged stimuli, low pass filter: 1,000 Hz—high pass filter 10 Hz and 6/oct.) at a stimulus intensity of 100 dBnHL (approximately 120-dB SPL). The amplitude (μv) of the first positive–negative peak responses—(p13-n23)—were recorded at baseline and after immersive *unidirectional visual motion exposure* (3 min) using optokinetic visual stimulation (VS) and Virtual Reality googles (VRg).

The cVEMP responses, pre and post VS, were normalized by the electromyographic activity and subsequently analyzed. Stimulation paradigm in Figure 1.

### 2.3 Visual stimulation paradigm

After electrode montage, a *unidirectional visual motion exposure* was delivered through a virtual reality headset. The headset holds the phone in front of magnified lenses. The device provides a 120 degrees field of view (ETVR headset, Amazon, N. D, Version 3.0).

Once the headset was placed, participants received the instruction to look straight ahead at the visual stimulus, which consisted of an optokinetic stimulus composed of black and white vertical stripes moving rightwards at a speed of 2.65 cm/s. This stimulus was accessed through a Smart Optometry app (Smart Optometry., N. D). Participants were instructed to normally blink but keep their eyes open during the whole duration of the visual stimulation. Visual stimulation was delivered for a total of 3 min, using a timer, however participants were not informed of the length of time. They were also instructed to adjust the headset for comfort and report any light leakage to ensure full immersion in the visual motion paradigm.

Visual stimulation was conducted immediately after the first baseline cVEMP, and a second cVEMP was performed immediately following VS.

### 2.4 Statistical analysis

Following a pilot study to previously explore threshold intensity cVEMP responses, this study only analysed responses at maximum intensity (100 dB nHL, ~120-dB SPL), as threshold responses were highly variable and inconsistent.

Data were logged in Excel. Statistical analyses were conducted using mixed-effects linear regression models in Stata v19. For cVEMP amplitude responses, the model included fixed effects and interactions between groups (control/VM), condition (baseline/post visual stimulation), ear (left/right). For Interaural asymmetry the model included fixed effects and interactions between groups (control/VM), condition (baseline/post visual stimulation), and asymmetry side (left/right). Model included a random effect of participant and allowed residuals to have different variances in each group.

For pairwise comparisons, non-parametric tests (Wilcoxon signed-rank test, Spearman’s rank correlations) were applied. Interaural amplitude asymmetry was calculated using the Jongkees formula, as follows:


$$\text{Asymmetry}\;(\%)={\frac{\mathrm{RA}-\mathrm{LA}}{\mathrm{RA}+\mathrm{LA}}}^{*}\;100$$

Where:

RA: Right ear cVEMP peak-to-peak amplitude (P13–N23), in microvolts (μv).

AL: Left ear cVEMP peak-to-peak amplitude (P13–N23) (μv).

## 3 Results

Table 1 shows the distribution of VM patients along with clinical details, including time since last episode, ongoing treatments, and symptoms responses to VS. All patients reported dizziness during VS, but only one required early discontinuation of the VS protocol (VS run for 2 min instead of 3).

**Figure 2:**
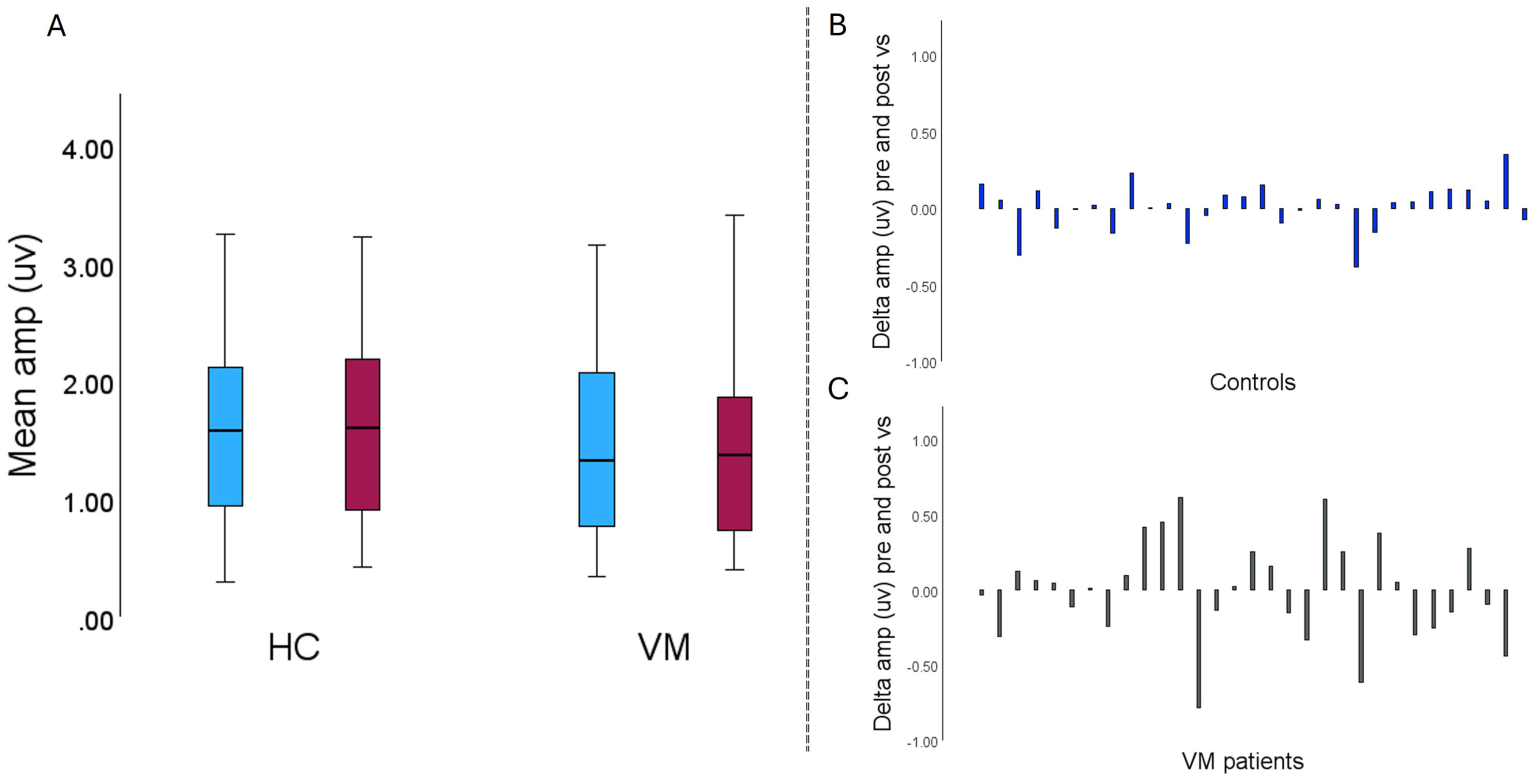
Mean and delta amplitude across healthy controls (HC) and vestibular migraine (VM) groups. **(A)** Clustered mean amplitude plot across healthy controls (HC) and vestibular migraine (VM) groups; at baseline (blue), and after visual stimulation (red). **(B)** Delta amplitude (μV): change in amplitude post-stimulation relative to baseline in HC and in **(C)** VM patients.

A Wilcoxon Signed Rank test, conducted to compare the difference between cVEMP amplitude in HC at baseline and after the VS exposure, indicated no significant differences between conditions scores in HC group, (*Z* = −0.741; *p* = 0.459, Figure 2). The same was observed when comparing cVEMP amplitude in VM at baseline and after the VS exposure, indicating no significant differences between conditions scores in VM group (*Z* = 0.031; *p* = 0.975, Figure 2).

**Figure 4:**
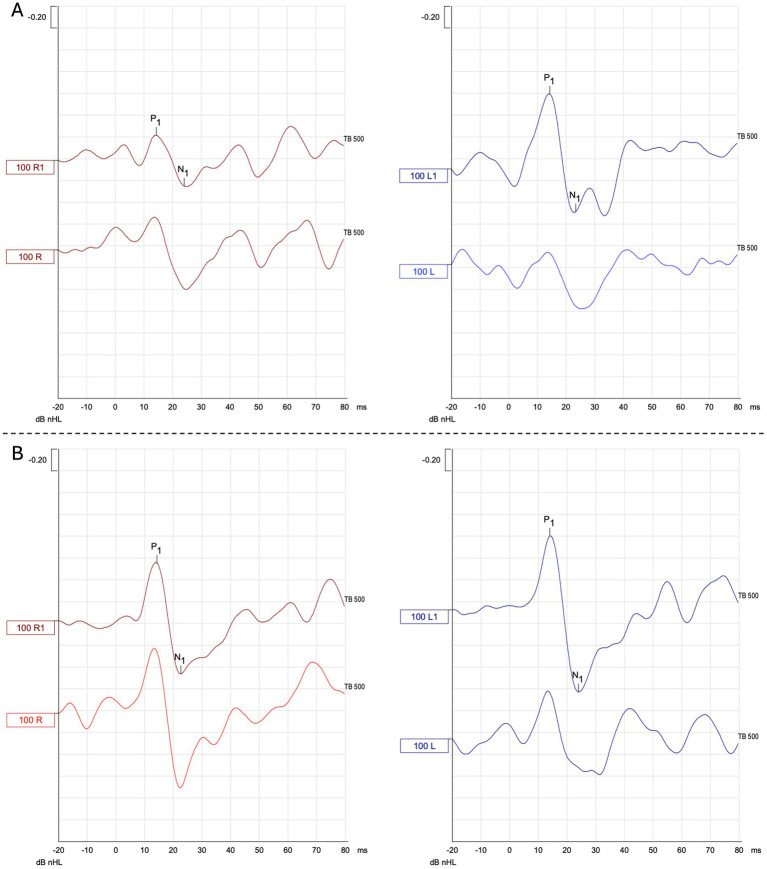
Representative AC - cVEMP waveform responses **(A)** before and **(B)** after VS in the VM group. dB nHL: Decibel normalized hearing level; TB: tone burst; 100 R1: right responses at 100 dB nHL; 100 L1: left responses at 100 dB nHL.

**Figure 5:**
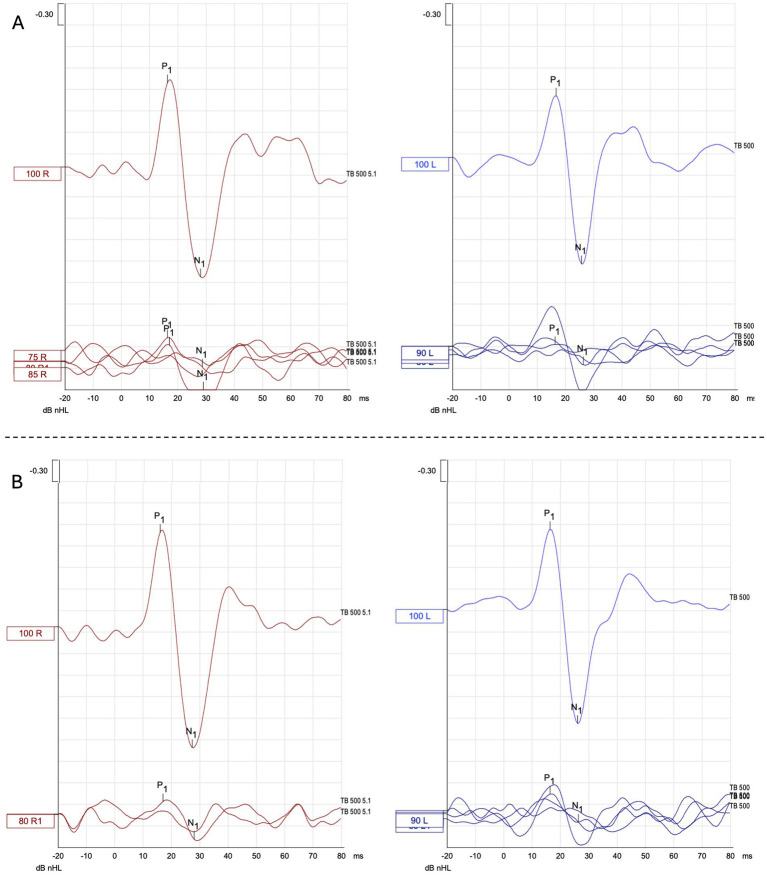
Representative AC - cVEMP waveform responses **(A)** before and **(B)** after VS in the HC group. dB nHL: Decibel normalized hearing level; TB: tone burst; 100 R1: right responses at 100 dB nHL; 100 L1: left responses at 100 dB nHL.

At baseline, the VM group exhibited slightly reduced response amplitudes relative to HC. A Mann–Whitney U test was conducted to assess the group distributions. The test statistic (*U* = 391.000) and standardized test statistic (*Z* = −0.872) revealed no significant difference between the two groups (*p* = 0.383), suggesting that their distributions were similar, with no evidence of significant shift in ranks or central tendencies.

Mixed linear model (MLM) type III tests indicated that there were no significant main effects of group, condition or ear on cVEMP amplitudes (all *p* > 0.05). Similarly, all interactions effects, including group x ear, condition x ear and the three-way interaction, were not statistically significant (all *p* > 0.05).

**Figure 3:**
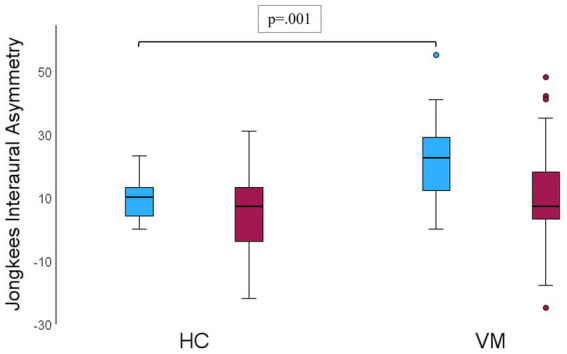
Jongkees interaural asymmetry across healthy controls (HC) and vestibular migraine (VM) groups; at baseline (blue), and after visual stimulation (red). VS, Visual stimulation. Error bars represent 95% CI.

Mixed linear modeling of interaural asymmetry (% asymmetry via Jongkees formula) revealed a significant main effect of group, *F*(1, 57.21) = 11.89, *p* = 0.001, with the VM group showing greater asymmetry overall compared to healthy controls (Figure 3). There was also a significant main effect of condition, *F*(1, 56.53) = 14.47, *p* < 0.001, indicating that asymmetry changed significantly from baseline to post-visual stimulation in both groups. However, the group × condition interaction was not significant, *F*(1, 56.53) = 1.57, *p* = 0.215, suggesting that the degree of change in asymmetry from baseline to post-stimulation was similar across groups (all *p* > 0.05) (Figure 3).

Typical cVEMP waveform responses before and after VS are shown for both the VM group (Figure 4) and the healthy control (HC) group (Figure 5).

Spearman’s correlations in VM group showed no significant associations between cVEMP delta amplitude (pre-post visual stimulation) and DHI (*ρ* = 0.015, *p* = 0.935), MSSQ (ρ = −0.272, *p* = 0.145), HIT-6 (ρ = 0.067, *p* = 0.727), HADS-D (ρ = −0.062, *p* = 0.745), or HADS-A (ρ = −0.209, *p* = 0.268). As expected, patients scored significantly worse on the DHI and MSSQ than healthy controls (All *p* < 0.001). The same tendency was seen also in HIT-6 and HADS scores (All *p* < 0.001) (Table 2).

**Table 2:** Results of the self-administered questionnaires.

	VM	HC
DHI, median (range)	48.0 (2–96)	0 (0–14)
MSSQ, median (range)	26.6 (5.5–54)	13.3 (0–42.6)
HIT-6, median (range)	60 (36–72)	40 (36–59)
HADS-depression, median (range)	6 (0–16)	1 (0–5)
HADS-anxiety, median (range)	8.5 (2–19)	4 (0–17)

DHI, Dizziness handicap inventory; MSSQ, Motion sickness susceptibility questionnaire; HIT-6, Headache impact test; HADS, Hospital anxiety and depression scale; VM, Vestibular migraine participants; HC, Healthy controls participants.

### 3.1 Sensitivity analysis

To assess the impact of outliers on the main asymmetry effect (Figure 3), a sensitivity analysis was performed. Outliers in VM group and their corresponding paired condition values (baseline vs. post-VS) in the same subject were temporarily excluded and a new MLM type III model run. The results revealed no changes in the main effect: significant main effects observed for group [*F*(1, 48.55) = 7.41, *p* = 0.009] and Condition [*F*(1, 47.44) = 16.79, *p* = <0.001], confirming that both factors significantly influenced asymmetry; and again no significant interactions were found, specifically Group × Condition [*F*(1, 47.441) = 1.939, *p* = 0.170]. These results confirm the robustness of the findings.

## 4 Discussion

We explored the effect of *Unidirectional Visual Motion Stimuli* delivered via virtual reality goggles on cVEMP amplitude responses of Vestibular Migraine patients and Healthy Controls participants, and we found that prolonged VS does not modulate cVEMP responses in interictal VM patients and therefore it cannot serve as a diagnostic biomarker. The observed interaural asymmetry behavior highlights a promising direction for investigating interaural and interhemispheric integration in VM.

To our knowledge, this is the first study to introduce VS to examine potential modulations of evoked myogenic responses in VM. Emerging evidence has identified an abnormal relationship between the vestibular and visual systems in VM patients (11), at least in relation to *perceptual thresholds* for motion detection (“Am I am moving?”) and for motion discrimination (“In which direction am I moving?”). Such findings have not to date been corroborated by paradigms that explore vestibular *objective* responses, which was the aim of this study. However, only maximum intensity responses were analyzed in this study (100 dB nHL, ~120-dB SPL) as threshold responses were highly variable and inconsistent. More importantly, this study analyzed changes in objective physiological responses, whereas earlier studies focused on perceptual thresholds—an important difference that may explain the differing results.

On the other hand, the results of this study to some extent align to those of a previous study that explored the presence of changes in vestibular excitability following a head-shaking paradigm (5), concluding that there was no effect on vestibulo-ocular reflex gain of patients with VM, at least in the inter-ictal phase. Despite key methodological differences across the two studies, both concluded that changes in vestibular afferent information (irrespective of applied stimulation paradigm) would not directly influence objective reflex response [VOR vs. vestibulo-collic reflex (VCR)]. This may relate to the use of stimuli that are insufficient to alter objective vestibular activity or maybe implies that such alteration can only be induced in patients with more severe symptom burden or amidst an acute episode of VM.

Of the available objective vestibular tests, both vHIT and VEMPs are perhaps the most practical to evaluate vestibular function objectively through measurement of reflex responses, namely the VOR and VCR. Both VOR and VCR are responses whose role is to maintain gaze stability and head position in relation to gravity, and as such, they correspond to primitive reflexes with very fast responses (23, 25, 26). While it has long been assumed that these responses do not involve cortical processing, more recent evidence challenges this assumption (27).

Taken together, present results would reaffirm the idea that the main differences between VM, healthy subjects and/or those with other diagnoses could lie more specifically in alterations associated with perception than in changes in physiological response that could be measured by objective tests of vestibular function. One might also assume that objective vestibular reflexes may be less heavily influenced by visual stimulation than perceptual vestibular function. As such, it may be necessary to explore new paradigms that more strongly influence motion perception and/or visual perception, perhaps through more powerful visual stimuli or immersive tests. This is not necessarily an easy task and has so far been one of the reasons why it has not been feasible to compare results across studies.

In a closer examination of the results obtained in this study, it is noteworthy that at baseline condition, patients with VM appeared to exhibit lower cVEMP amplitudes responses than healthy controls. This has been reported previously (28, 29) using comparable sample sizes. Our study demonstrated the same general trend; however, in our sample this result did not reach statistical significance.

We also found that the asymmetry ratio between ears, calculated trough the Jongkees formula, was significantly greater in the VM group than in healthy controls. This pattern has been previously observed in adults (29) and similarly described in the ocular vestibular myogenic potential responses (oVEMP) in children (30). Our results are consistent with this previous evidence, highlighting percentage asymmetry as a potentially valuable parameter for further investigation in the search for early VM biomarkers. Interestingly, the interaural asymmetry observed in VM patients at baseline, decreased following prolonged VS. This effect was independent of stimulus direction, asymmetry side, or any other interactions.

These findings suggest that if visual stimulation modulates cVEMP amplitude—regardless of the effect’s direction—the asymmetry observed in VM may not reflect a “true” peripheral asymmetry but rather and altered central processing and/or poor integration of interaural signals. Our results support baseline cVEMP asymmetry as a potential central feature of VM and provide a basis for further exploring a potentially disrupted central integration in these patients (2).

As was previously elaborated, it is known that the cVEMP response relies on central integration of peripheral vestibular signals, ultimately generating an ipsilateral inhibitory response (23, 25, 26). Moreover, a growing body of evidence suggests that one of the key elements of VM is the difficulty in integrating sensory signals, particularly those involved in spatial orientation and balance (2, 31, 32). We hypothesize that this impaired multisensory integration may also extend to the brainstem level and could be greatly contributing to the higher asymmetries observed in VM group, which may in turn, be associated with their symptomatologic presentation.

### 4.1 Limitations and strengths

Although all the patients were in the active disease stage, that is, having had at least one attack in the last year, some were undergoing pharmacological treatment to control the intensity of their symptoms, and their responses may not be representative of uncontrolled migraine status. Although it would be ideal to assess patients with VM during the acute or ictal phase, this is operationally quite difficult, due to the severity and nature of symptoms.

Although the generalizability of these findings may be limited by single-center recruitment and relatively small sample size, the study’s rigorous design and careful sample selection—restricted to patients with a definite VM diagnosis made by neuro-otologist specialists according to Barany Society criteria—supports the internal validity of the results. This means that while caution is needed when extending the findings to other settings or more diverse population, the study provides a reliable representation of the core characteristics of the broader VM population.

## 5 Conclusion

In this study, prolonged unidirectional visual stimulation did not result in significant modulation of cVEMP responses in an interictal phase in VM patients. These findings suggest that cVEMP amplitude induced by this specific visual stimulation paradigm is unlikely to serve as a reliable diagnostic biomarker for VM. However, given the methodological limitations and the relatively small sample size, the present results should be interpreted with caution and considered preliminarily as hypothesis-generating, highlighting the complexity of probing visuo-vestibular dysfunction in VM, rather than interpreting them as definite evidence against the diagnostic or pathophysiological relevance of visual-vestibular interaction paradigms in this condition. The observed vestibular asymmetry at the group level points toward potential alterations in interaural and interhemispheric sensory integration in VM. Future studies incorporating larger cohorts will be essential to further characterize visuo-vestibular dysfunction and to clarify its potential diagnostic relevance in VM.

### 5.1 Clinical implications

- cVEMP amplitude modulation by visual motion is unlikely to serve as a useful bedside biomarker for vestibular migraine in acute or emergency settings, as responses do not reliable distinguish Vestibular Migraine patients from Healthy Controls.
- Interaural asymmetry may better reflect altered visuo-vestibular sensory processing in VM, supporting further investigation of asymmetry-based metrics in clinical assessment.
- Persistent symptom burden despite normal brainstem reflex amplitudes reinforces the central (sensory integration) origin of vestibular migraine, highlighting the need for diagnostic approaches that probe higher-order multisensory processing rather than peripheral vestibular function.

## Data Availability

The raw data supporting the conclusions of this article will be made available by the authors, without undue reservation.
